# Prevalence and factors associated with bullying phenomenon among pre-adolescents attending first-grade secondary schools of Palermo, Italy, and a comparative systematic literature review

**DOI:** 10.1186/s13052-022-01245-2

**Published:** 2022-04-04

**Authors:** Claudio Costantino, Walter Mazzucco, Francesco Scarpitta, Gianmarco Ventura, Claudia Marotta, Stefania Enza Bono, Evelina Arcidiacono, Maurizio Gentile, Pierfrancesco Sannasardo, Carlo Roberto Gambino, Claudia Emilia Sannasardo, Carlotta Vella, Francesco Vitale, Alessandra Casuccio, Vincenzo Restivo

**Affiliations:** 1 Department of Health Promotion, Maternal and Infant Care, Internal Medicine and Medical Specialties “G. D’Alessandro”, Section of Hygiene, University of Palermo, Via Del Vespro 133, 90127 Palermo, Italy; 2 Cincinnati College of Medicine, Department of Pediatric – Division of Biostatistics and Epidemiology, Cincinnati Children’s Hospital Medical Center, Burnet Ave 3333, Cincinnati, OH 45229 USA; 3 Regional Educational Authority of Sicily, Via G. Fattori 60, 90100 Palermo, Italy; 4 Local Health Authority of Palermo, Via G. Cusmano 24, 90141 Palermo, Italy

**Keywords:** Bullying, Systematic literature review, Bullying prevalence, Observers, Pre-adolescents

## Abstract

**Background:**

Bullying is recognized as one of the most significant social and health problems in the school environment for children and adolescents. In Italy, bullying involved 2 in 10 kids between 11–17 years that referred to have been bullied two or more times in a month. In Sicily, the estimated prevalence of children aged 11 to 15 that suffered at least one act of bullying in the last two months was 14% in 2011.

**Methods:**

A questionnaire consisting of 30 items investigating physical, verbal and indirect bullying, observers of bullying, resiliency, and prosociality was administered to preadolescents of ten first-grade secondary schools within the Palermo Province in order to analyze prevalence and factors associated with bullying phenomenon. Also, a systematic literature review (SLR) analyzing manuscripts that reported prevalence of the bullying phenomenon worldwide was conducted.

**Results:**

*Survey*: a total of 867 students, belonging to 35 s and 31 third classes of ten different schools in Palermo, Italy, were recruited in the survey. The values of physical bullying are included between the 4% of the single question method and the almost forty percent detected by the score of 7 method. Verbal bullying oscillates between 15.9% and 66.3%. Observers average values varies from 15.8% to 47.5%.

*SLR*: the estimated prevalence showed a considerable fluctuation. The occurrence of the bullying phenomenon was low in some Northern European countries, while in Anglo-Saxon countries it affected over a quarter of the middle school student population (28% in Maryland, USA and 21% in the UK).

**Conclusions:**

The prevalence of the bullying phenomenon recorded by this survey with the three different methods used is similar to observations in international literature. In the Sicilian context, a higher prevalence of bullying phenomena was observed in pre-adolescents attending major classes and in schools with lower socio-economic index. Though it remains difficult to obtain univocal data that clarifies the prevalence of different type of bullying, the continuous investigation of prevalence and factors associated with the phenomenon is a necessary starting point to introduce interventions and preventive measures in Public Health programs.

## Background

Bullying is recognized worldwide as one of the most significant social and health problems in the school environment for children and adolescents [1]. In recent decades, bullying has gained a growing interest in public health, catalyzing many efforts both in research and in action [2, 3]. Currently, this marked interest on the topic has not been followed by a universally recognized agreement on their definition.

Taking into account the local peculiarities of bullying, the global burden of the phenomenon is hard to estimate [4].

In the early nineties of the last century, based mainly on the works of Olweus [5–8], and in the first decade of this century, referring to the works of Farrington [9–12], a more scientific approach in the evaluation of bullying emerged.

In particular, the most punctual and universally accepted definition of bullying, that identified intentionality, duration over time and asymmetry in the relationship as the main elements of bullying phenomenon, was attributed to the work of Farrington et al. [9].

On the other hand, the implementation of surveys that allowed a detailed analysis of the phenomenology of bullying and the characteristics of its main actors (bullies, victims and observers) was due to the Norwegian school of Olweus and colleagues [5]. Victims can present the most disparate characteristics of "diversity": ethnicity, obesity, sexual habits or, more generally, elements that characterize only a small minority of subjects belonging to a group [13, 14]. More recently, the "cyber-bullying" phenomena has spread, consisting of episodes of bullying perpetrated through electronic tools (computers, smartphones) and favored by the growing access to the internet even among young and very young people [12, 15, 16].

In Italy, bullying involved 2 in 10 kids between 11–17 years that referred to have been bullied two or more times in a month [17].

Moreover, in Sicily, the first Italian region by territorial extension and the fourth most populous, the estimated prevalence of children aged 11 to 15 that suffered at least one act of bullying in the last two months was 14% in 2011 [18].

Prevalence data and factors associated with bullying phenomenon obtained in a survey conducted by the Bullying in Sicilian Schools (B.I.A.S) working group among students attending first-grade secondary school in the metropolitan area of Palermo, the largest city in Sicily (Italy) were investigated.

Moreover, we aimed to analyze the prevalence of bullying that has emerged in other studies conducted by groups and by national or supranational institutions, also to compare different strategies used in the detection of bullying phenomena.

## Materials and methods

### Questionnaire

During the school year 2017/18 the BIAS (Bullying in Sicilian Schools) working group carried out a survey among students attending ten school institutions in the City of Palermo, Sicily [19], in order to estimate the prevalence of bullying in the pre-adolescent age group.

The City of Palermo is the most populous Sicilian city. The study population included all the 22,455 school-aged children, attending one of the 58 secondary first-grade schools of Palermo, Italy. A two-stage cluster sampling process was performed. Based on the 14% bullying among Sicilian students (prevalence), with a 99.9% desired level of significance and an average number of 20 students per class, the minimum study population needed was 555 students. Schools were considered as primary units of the sampling, while classes as secondary units.

The schools, based on sociodemographic criteria, were categorized into three levels – high (A), intermediate (B), and low (C) – in accordance with the neighborhood socioeconomic index (SEI), based on the logarithm of the median household income, the proportion of adults aged 25 years or older with a high school diploma or college degree, and the proportion of people employed. Then, at least three schools and a minimum of 4 classes in each school from each level were selected.

After obtaining written consent from their parents, an anonymous questionnaire was self-administered by the students from December 2017 to February 2018, using an open access web-based platform (Google® Forms) to evaluate the baseline prevalence of bullying from the students’ perspective. The questionnaire consisted of an introductive section on socio-demographic data (gender, age, nationality, school institution and class attended), followed by 30 items investigating the six main areas of practical interest and research on bullying: 1) physical bullying, 2) verbal bullying, 3) indirect bullying, 4) observers, 5) resiliency, and 6) prosociality (five questions for each area) [19, 20].

All 30 questions are based on a Likert Scale from 1 to 5 as follows [19]:

1. Never
2. Rarely (only once or twice)
3. Occasionally (three to six times)
4. Often (about once a week)
5. Very often (several times a week)

An open-ended section to freely express thoughts about 1) the content of the questionnaire and 2) bullying in general was included at the end of the questionnaire. For each answer, a score between one (never) and five (very often) was assigned.

The score was then used to detect the baseline level of bullying with the following three methods: 1. *Sentinel question method*, where the presence or absence of bullying was investigated through “yes” answers to the most significant questions in an area. The responses very often, often, and occasionally were considered affirmative answers. 2. *The five-question method,* which considered bullying to be present whenever the student answers yes (i.e. occasionally, often, or very often) to at least one of the items in the survey area. 3. *The score of seven method*, where the answers to each question were scored and added and the presence or absence of bullying was then determined, while the value of seven was considered the cutoff (i.e. the respondent could answer occasionally to at least one of the questions in an area).

The sentinel questions for the six areas were: 1. “Since I started first-grade secondary school, another boy or girl has hurt me alongside other boys or girls” (physical bullying); 2. ““Since I started first-grade secondary school, another boy or girl has insulted me (even on online platforms)” (Verbal bullying); 3. “Since I started first-grade secondary school, another boy or girl has turned another boys or girls against me (even on online platforms)” (Indirect bullying); 4. “Since I started first-grade secondary school, I saw a friend insulted by another boys or girl (even on online platforms), but I preferred to mind my own business” (Observers); 5. “Since I started first-grade secondary school, if I am bullied, I rebel “ (Resiliency); 6. “Since I started first-grade secondary school, I was bullied and I talked about what happened with the teacher (Prosociality).

Cronbach’s alpha was carried out in order to evaluate the estimated reliability of the questionnaire.

In this study the Cronbach’s alpha for physical, verbal, indirect bullying, and also for observers, prosociality and resiliency were calculated and corresponded to 0.86, 0.84, 0.86, 0.88, 0.87 and 0.86 with an adequate reliability of the test.

Data obtained were exported into an electronic database created by Excel 16.0 software and analyzed using STATA14® software.

### Systematic literature review (SLR)

A systematic literature research of the main manuscript that reported the prevalence of the bullying phenomenon published between January 2011 and February 2019 was conducted. The research was conducted on electronic databases including PubMed/MEDLINE, SCOPUS, EMBASE, ISI Web of Science, Google Scholar as well as the grey literature. In details, the following search terms were used: *((bullying AND prevalence)) AND ("2011/01/01"[Date—Create]: "2019/02/28"[Date – Create])).*

Inclusion criteria:

- Type of publication: original scientific articles or reports;
- Publication of the source from January 1, 2011 to February 28, 2019;
- Language of publication: English;
- Area of publication: worldwide;
- Full text available online.

The research was set to achieve a representativeness of all continents, focusing mainly on the European one.

Exclusion criteria:

- Publication of the paper or report prior to January 1, 2011 (with the exception of works concerning countries for which no more updated data were present);
- Reviews and meta-analysis; works not published in English;
- Inclusion of subjects under study aged less than ten years or over seventeen;
- Studies still in progress as of February 28, 2019;
- Lack of the definitive prevalence data specified in the results;
- Lack of a defined and explicit protocol describing how the prevalence data had been obtained;
- Lack of representativeness of the subjects in relation to the demographic characteristics of the study population and the territory to which they belong;
- Tangible lack of relevance of the papers / reports with the aim of this study, after reading their abstracts.

The working group may have further applied additional exclusion criteria not previously considered during the reading of full-text contributions. The results were then extrapolated from the articles and reports found, summarized and, finally, organized in a comparison table with the results of this survey.

### Statistical analysis

All categorical variables were reported as absolute and relative frequencies (percentages). Chi-squared tests (with Fisher’s correction where appropriate) were used to compare categorical variables and comparative analyses performed by school socioeconomic index. Quantitative variables were normally distributed and summarized as means with their standard deviations. Differences in means were compared with the Student t-test for a paired sample. All the variables found to have an association with bullying phenomenon ≤ 0.20 (any form of physical, verbal, indirect bullying or observer role evidenced with the five-question method) in the univariate analysis were included in a backward stepwise logistic-regression model. Adjusted OR (adj-OR) with 95% confidence intervals (95% CIs) were calculated for the variables retained in the final model. The significance level fixed for the whole analysis was 0.05, two-tailed.

## Results

### Survey results

A total of 867 students, belonging to 35 s and 31 third classes of ten different schools in Palermo, Italy, were recruited in the survey. Among students, 51% (*n* = 444) were females and 49% (*n* = 423) were males. Only 2% (*n* = 20) of respondents were of foreign nationality. The average age of the entire sample was 12.3 years (± SD = 1.23) (data not shown).

Table 1 shows the prevalence of the main types of bullying investigated (physical, verbal and indirect), according to the three used methods of analysis.

**Table 1:** Prevalence of bullying by comparing the three methods selected and average values of bullying phenomenon in the study population (*n* = 867)

	**Methods**	**Prevalence (%)**
**Physical**	Single question	35 (4)
	Five questions	241 (27.8)
	Score of 7	346 (39.9)
**Verbal**	Single question	138 (15.9)
	Five questions	370 (42.7)
	Score of 7	575 (66.3)
**Indirect**	Single question	105 (12.1)
	Five questions	295 (34)
	Score of 7	431 (49.8)
**Observer**	Single question	137 (15.8)
	Five questions	288 (33.2)
	Score of 7	412 (47.5)
**Average Bullying values**	Single question	(11.8)
	Five questions	(34.3)
	Score of 7	(50.9)

The values of physical bullying are included between the 4% of the single question method and the almost forty percent detected by the score of 7 method. Verbal bullying oscillates between 15.9% and 66.3%. Observers average values varies from 15.8% to 47.5%.

Finally, the indirect one increases from the 12.1% of the single question to the 49.8% derived from the score of 7 methods. Finally, the average value of bullying obtained considering the mean prevalence of physical, verbal, indirect bullying and observers was 11.8% for the single question method, 34.3% for the five questions method, and 50.9% with the score of 7 method.

In Table 2 data on attitudes and roles of the actors involved in bullying episodes are shown. Attitude of prosociality started from a minimum of 12.5% (single question) up to 89.2% (score of 7). Resiliency showed a range of about thirty percentage points (from 37.8% to 68%).

**Table 2:** Prevalence of anti-bullying attitudes and roles by comparing the three methods selected among the study population (*n* = 867)

	**Methods**	**Prevalence (%)**
**Prosociality**	Single question	108 (12.5)
	Five questions	709 (81.8)
	Score of 7	773 (89.2)
**Resiliency**	Single question	328 (37.8)
	Five questions	479 (55.2)
	Score of 7	590 (68.0)

Table 3 reports the univariate (Crude OR) and multivariate (Adj-OR) analysis between the bullying phenomenon prevalence (physical, verbal, indirect bullying or observers) correlated with sociodemographic factors (gender, class, nationality and school SEI) of the population in study.

**Table 3:** Univariate (Crude-OR) and multivariate (Adjusted-OR) analysis of factors associated with bullying phenomenon (average physical, verbal, indirect bullying or observer prevalence with the five-question method) in the study population (*n* = 867)

Factors associated with bullying phenomenon						
	**Crude-OR**	**CI 95%**	***p*-value**	**Adj-OR**	**CI 95%**	***p*-value**
Age (for each additional year)	0.98	0.68–1.41	0.18	0.96	0.76–1.21	0.73
Gender (male vs female)	1.12	0.65–1.89	0.36			
School year (Third Vs Second)	1.89	1.42–2.87	< 0.01	1.76	1.21–2.58	< 0.01
Nationality (Foreign vs Italian)	1.56	0.52–2.34	0.55			
School SEI (for decreasing socio-economic index)	1.24	1.12–1.69	< 0.05	1.21	1.06–1.53	< 0.05

The bullying phenomena resulted more frequently in higher school classes, in particular (third vs second year of study course). A significant higher prevalence of bullying was observed in students attending third level classes (OR 1.76; CI 95% 1.21—2.58; *p*-value < 0.01) and in students attending schools with lower socio-economic index (OR 1.21; IC 95% 1.06—1.53; *p*-value < 0.05).

### Systematic literature review (SLR)

**Fig. 1:**
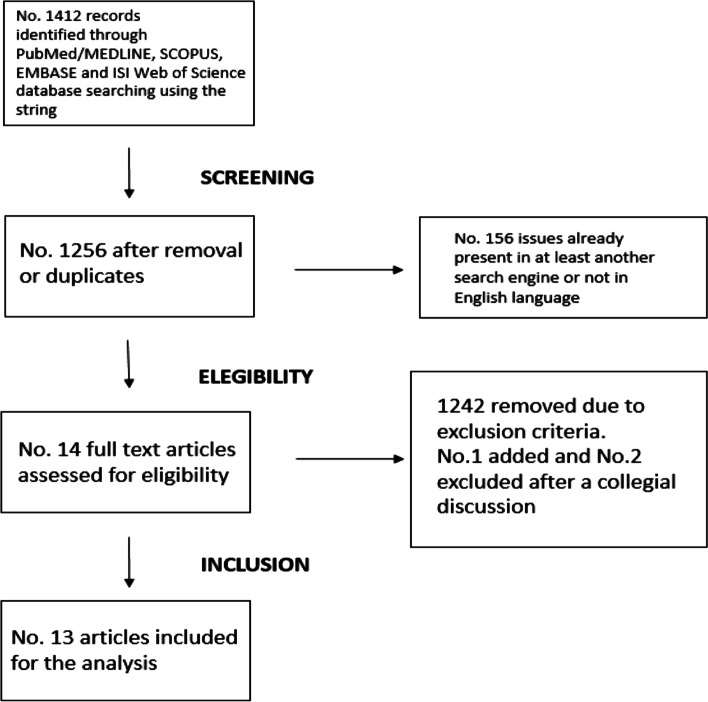
Flow systematic literature review diagram of the manuscripts reporting the prevalence of the bullying phenomenon

A total of over 1,400 scientific articles emerged from a first phase of bibliographic research. In addition, national, continental and global data-based reports, published on institutional websites, were considered. During the screening phase, 1,256 manuscripts remained after removal of duplicates and manuscripts written in languages other than English (Fig. 1). During the eligibility phase, 1,242 articles were removed due to exclusion criteria (Fig. 1).

Specifically, during this phase, one manuscript was added and two were excluded from the review after collegial discussion among the working group. At the end of the process, thirteen studies were selected for comparison (Fig. 1).

The eligible manuscripts (Table 4) were from four different continents (America, Asia, Europe and Oceania). As far as the African continent is concerned, no surveys referring to individual states have been found, but only data limited to individual urban areas or aggregate data of supranational reports. The selection of the article covers a period of eight years, from 2011 to 2019 [21–33]. After a collegial discussion involving the entire working group, we decided to include two works concerning Tajikistan and the Republic of Macedonia, respectively, as the last ones available, in a chronological sense, to investigate the phenomenon of bullying in these specific areas. Also, two surveys included first (Boston College, 2011, and London School of Economics and Political Science, 2014) were subsequently excluded after a revaluation by the working group considering them inhomogeneous toward guaranteeing comparability with the others selected studies.

**Table 4:** General characteristics and prevalence of bullying observed in the studies selected during the SLR

Country	Year	Authors	Title	Detection mode	Sample and age class	Bullying Prevalence
Denmark, Finland, Greenland, Iceland, Norway, Sweden	2019	Amarsson et al. [21]	Cyberbullying and traditional bullying among Nordic adolescents and their impact on life satisfaction	HBSC 2013/2014 (single item: “How often have you been bullied at school in the past couple of months?”)	32,210 students (11, 13 and 15 y.o.)	Norwegian Males: 2.7–7.8% Females: 3.1–5.2% Danish M: 2.7–8.0% F: 2.7–7.3% Finnish M: 6.4–12.6% F: 6.5–8.2% Greenlander M: 13.0–26.4% F: 13.7–18.6% Icelandic M: 1.4–6.9% F: 0.3–5.3% Swedish M: 2.1–3.5% F: 2.1–5.7%
Iceland	2018	Garmy et al. [22]	Bullying in School-aged Children in Iceland: A Cross-sectional Study	HBSC 2013/2014 in Iceland	11,018 students (11, 13 and 15 years old)	5.6%
Australia	2017	Thomas et al. [23]	Prevalence and correlates of bullying victimization and perpetration in a nationally representative sample of Australian youth	Single item: “In the past 12 months, how often were you bullied or cyberbullied by another person or group of young people?”	2,967 (11–14 y.o.)	15.5%
China	2017	Han et al. [24]	School Bullying in Urban China: Prevalence and Correlation with School Climate	Ten items on physical, verbal bullying and observers phenomena	1,020 middle school students	Victims: 26.10% Observers: 28.90% (aggregated data)
United Kingdom	2017	Muijs et al. [25]	Can schools reduce bullying? The relationship between school characteristics and the prevalence of bullying behaviors	For students: The Olweus Bully‐Victim Questionnaire (OBVQ). For teachers: 65 items-questionnaire based on Kyriakides (2014) scales	Survey conducted in 35 primary schools (1,411 last year's students and their 68 teachers)	Victims: 21% Actors: 11%
United Kingdom	2017	Bevilacqua et al. [26]	The role of family and school-level factors in bullying and cyberbullying: a cross sectional study	Gatehouse Bullying Scale (12 items)	6,667 students from 40 middle schools	Male: 9.99% Female: 13.61%
USA (Maryland)	2017	Waarsdorp et al. [27]	Ten-Year Trends in Bullying and Related Attitudes Among 4th- to 12th-Graders	10 years survey; anonymous and 13 items questionnaire	246,306 students in 109 schools of Maryland	28.5% in 2005 13.4% in 2014
Vietnam	2017	Le et al. [28]	Temporal patterns and predictors of bullying roles among adolescents in Vietnam: a school-based cohort study	Two anonymous questionnaires; single item: “How often have you been bullied in any way during the last six months?”	1,424 middle and high school students	48.3%: not involved at all; 22.7% victims only; 6.9% bullies only; 22% bully-victims After six months: 62.1% not involved; 17.6% victims only; 4.7% bullies only; 15.5% bully-victims
Spain	2016	Sanchez-Quelia et al. [29]	Trend analysis of bullying victimization prevalence in Spanish adolescent youth at school	Based on HBSC 2014 in Spain	15,728 (11–12 y.o.)	5.5% (reported) 23.6% (observed)
U.S.A	2015	Huang et al. [30]	The impact of definition and question order on the prevalence of bullying victimization using student self-reports	Anonymous, online, 100 items-survey	17,301 students attending 119 high schools	Bullying (overall): 9.84% Vs 13.53% cases Vs controls
Italy	2014	National survey [31]	Health Behaviour in School-aged Children	A single item within a broader nationwide questionnaire, mainly focused on habits and lifestyles	4,072 (11–15 y.o.)	14%
Macedonia	2007	WHO-Unicef-CDC [32]	Global school-based student health survey (GSHS)	Single item: “During the past 30 days, on how many days were you bullied?”	2,114 students (13–15 y.o.) in 30 schools	10.1%
Tajikistan	2006	WHO-Unicef-CDC [33]	Global school-based student health survey (GSHS)	Single item: “During the past 30 days, on how many days were you bullied?”	9,714 students (13–15 y.o.) in 99 schools	7.4%

The evaluation of bullying prevalence was conducted among the pre-adolescents and the adolescents attending first-grade secondary schools through self-compiled questionnaires, in paper or online anonymous forms.

None of the selected works included a face-to-face interview conducted by an adult. With regard to the number of questions, they varied from a single question, included in more structured works, to dedicated questionnaires consisting of several questions.

The time window investigated ranged from thirty days prior to completing the questionnaire (two studies), up to two, six or twelve months before (one study). Other works analyzed the prevalence of bullying during the whole school year, regardless of the number of months considered.

Four of the European publications reported data extrapolated from the last Health Behavior in School-aged Children (HBSC) survey. At least three of the selected works explicitly investigated the area of electronically perpetrated bullying (cyber-bullying). Nearly all the studies analyzed the relation between the victim and the bully.

The sample size of the enrolled students was extremely variable in the different surveys, ranging from a few hundred to over two hundred thousand children involved.

Even the estimated prevalence showed a considerable fluctuation: the occurrence of the bullying phenomenon was low in some Northern European countries (for example, Iceland and Sweden, which reported prevalence between 0.3 and 5.7%) [21, 22], while in Anglo-Saxon countries it affected over a quarter of the middle school student population (28% in Maryland, USA and 21% in the UK) [25–27, 30]. Finally, among the selected studies, there was an outlier value of close to a third of the interviewees (29% declared to have witnessed bullying attitudes) recorded in China [24].

## Discussion

In line with some of the international studies included in this SLR, a single item prevalence of the bullying phenomenon (10.7%) has been recorded by this survey [21, 23, 32, 33].

Moreover, similar trends in augmented prevalence of the bullying phenomenon have been reported through multiple item questionnaires both in this study (34.8%) and other experiences [24, 25, 27].

Probably, when facing the argument on the surface, this data could be interpreted as a former reticence of adolescents to discuss the bullying phenomena, while a latter good attitude to talk about it could be recalled, whereas the topic is more deeply discussed (i.e. using multiple basic questions).

So, on the one hand, “single item” prevalence might underestimate the bullying phenomenon (too specific question – 10.7%), but, on the other hand, “score of 7” prevalence might overestimate the phenomenon (too sensitive tool – 52%).

It’s reasonable to think, and we used the present methods to evaluate factors associated with bullying phenomenon in the present study, that the “five question” prevalence method represents the one next to the reality at most (34.8%).

In the national survey based on the “health behavior in school-aged children – HBSC” questionnaire analyzed the topic throughout a single-item question and the prevalence was similar to what observed in our survey with the same method, probably largely underestimating it like reported in Spain [21, 22, 29, 31].

At the same time, where the bulling phenomena were investigated with multiple items survey, the prevalance of bullying observed was similar to what reported in Sicilian survey with “the five-question” or “the score of 7” methods [24, 25, 27, 30].

Furthermore, this study draws attention to some important points for reflection.

First of all, it sheds light on the usually neglected figure of the observers of bullying phenomena, that usually contributed to the perpetration of bullying phenomenon as previously stated [24]. Secondly, it reaffirms the presence of a growing trend, with regard to the children’s age, proportional to the degree of risk of being involved in bullying. We have documented how the phenomena more easily develops in the third classes than the latter ones, probably suggesting the presence of dynamics which are inveterate among the older children.

Moreover, our study reaffirm the role of preadolescents students prevention and contrast of bullying with resilience and prosociality that reveal higher prevalence with all the methods used and independently from other sociodemographic factors..

Bullying, in this direction, also follows the development of other risk situations, such as the beginning of the cigarette smoking habit and the voluptuous use of alcohol, typical of the adolescent or youth ages [34–38].

Another element to take into consideration is the children’s school attendance falling within the territory belonging to the "C band". Similar results were previously observed in the B.I.A.S. study, examining prevalence and characteristics of the bullying phenomenon from the teachers’ point of view [39].

Specifically, affective-relational discomfort, character/natural disposition and socio-cultural context were reported by the teaching staff to be the main factors associated with bullying [39].

Similarly, in the present study we observed as a relevant risk factor for the development of dynamics favouring episodes of bullying, probably due to “disadvantaged” familial and social contexts, in students attending schools belonging to “C” socioeconomic index. Low socioeconomic background of families might have influenced children’s involvement in bullying and victimization in several ways. Parental educational level reflects intellectual resources, general and specific knowledge, norms and values, literacy, and problem-solving skills; all aspects that could be related to child-raising behaviour and, consequently, to children’s development of social skills and coping strategies. Even in this case, bullying does not present a dissimilar trend when related to other indicators and / or effects of social discomfort among adolescents [40, 41].

The B.I.A.S. study also gave the opportunity to the adolescents to freely express through an anonymous online questionnaire, avoiding a selection bias of victims or of bullies [42]. Moreover, the bullying phenomena were analyzed using three different methods (single question; five questions; score of seven) which tried to estimate this social problem more accurately.

Moreover, our findings highlighted no significant differences between gender, nor between Italian and foreign students, although it has not been possible to clarify this last aspect, given the imbalance between the two sample sizes.

In addition, our study allows confirming the initial hypotheses of the B.I.A.S. working group regarding the analysis of the bullying phenomenon, which could fluctuate according to the used method. In particular, what emerged is how the use of a single item in the detection of the prevalence of bullying tends to underestimate the extent of the phenomenon, while, globally considering the answers provided for each area of investigation, the values found seem to describe class dynamics more faithfully.

Specifically, a more sensitive method of analysis addresses prevalence values up to 5 times higher than those that prefer greater specificity, such as the ones built on a single item. Similarly, protective and preventive attitudes towards bullying and the key role played by observers also emerged with greater force. In this direction, similar differences can be found in an external consultation, even in surveys carried out in other areas of the globe. If, among the studies selected in this review, we separately consider the studies that exploit a single question to detect the prevalence of bullying and those that rely instead on a structured questionnaire, we note that the former gave a prevalence datum of around 10% globally. This threshold (of about 18%) instead appears decidedly more worrying (and probably more adherent to reality) when we refer to the surveys built with a battery of questions that investigates more aspects of life and the relationships of young people.

In line with some of the international studies included in this SLR, a single item prevalence of the bullying phenomenon (10.7%) has been recorded by this survey [21, 23, 32, 33]. Furthermore, similar trends of higher prevalence of the bullying phenomenon have been reported through multiple item questionnaires (and detection methods) both in this study (34.8%) and other experiences [24, 25, 27].

Probably, when facing the argument on the surface, this data could be interpreted as a former reticence of adolescents to discuss the bullying phenomena, while a latter good attitude to talk about it could be recalled, whereas the topic is more deeply discussed (i.e. using multiple basic questions).

So, on the one hand, “single item” prevalence might underestimate the bullying phenomenon (too specific question – 10.7%), but, on the other hand, “score of 7” prevalence might overestimate the phenomenon (too sensitive tool – 52%). It’s reasonable to think that the “five question” prevalence method represents the one next to the real phenomenon at most (34.8%).

A previous SLR conducted by the B.I.A.S. working group tried to investigate the association between family environment and the bullying phenomenon among school-age children in order to find some determinants of interest [42]. Despite the fact that none of the included studies have made it possible to identify determinants that can directly affect a greater or lesser probability of incurring bullying among school-age children, SLR findings could suggest a connection with some determinants, such as generalized anxiety, low self-esteem, peer relationship problems, hyperactivity and social exclusion, thus providing a paint of the psychological profile of the bully and/or victim [43, 44].

In any case, by additionally adhering to institutional data, which instead refer to the values found by this working group, it is evident that bullying in Italy still represents a phenomenon of wide scope, characterized by territorial peculiarities, with a higher frequency than what it reported in neighbouring countries or in areas with similar socio-demographic characteristics.

As reported in the present SLR, only few studies (*n* = 13) worldwide accurately analyzed the prevalence of bullying in the last decade despite it being considered an important public health problem universally.

The present study contributed to accurate evaluation of the real prevalence of bullying in school-aged children in one of the most populated Italian cities. The collection of data in other prevalence studies (such as during the “health behavior in school-aged children” study) was limited to one or two non-specific questions and did not analyze the real impact of the phenomenon. The encouraging results obtained in a more accurate evaluation of the bullying prevalence and in the definition of factors associated with higher bullying episodes could help public health authorities in organizing dedicated interventions in a school context [20, 39]. The main limitation of the BIAS study is the small but still representative number of the participants.

Using the same methods, further analysis could be conducted within the national and/or international context, in order to increase the representativeness and to evaluate the reproducibility of the present experience.

## Conclusions

The findings provided by this study suggest that, even if we aggregate the results coming from different areas of the globe, it remains difficult to obtain univocal data that clarifies the real extent of the bullying phenomenon. The difficult comparability is given by the heterogeneity of the detection methods, of the sample numbers and, not least, of the social, economic and cultural characteristics that permeate the environments in which the interpersonal relationships of children intertwine.

Nevertheless, investigating the different prevalence of perceived bullying phenomena in school environments all over the globe is a necessary starting point to introduce tailored corrective actions of Public Health, in order to improve the psychosocial well-being of the adolescents and to promote healthy social relationships between them, during such a critical phase of their inner evolutionary growth.

## Data Availability

Data and materials collected are available on reasoned request to the Authors.

## Abbreviations

BIAS: Bullying In Sicilian Schools
SLR: Systematic Literature Review
SEI: Socio Economic Index
